# Effects of a New Bioceramic Material on Human Apical Papilla Cells

**DOI:** 10.3390/jfb9040074

**Published:** 2018-12-16

**Authors:** Diana B. Sequeira, Catarina M. Seabra, Paulo J. Palma, Ana Luísa Cardoso, João Peça, João Miguel Santos

**Affiliations:** 1 CNC-Center for Neuroscience and Cell Biology, University of Coimbra, Coimbra 3004-504, Portugal; disequeira@gmail.com (D.B.S.); cseabra@cnc.uc.pt (C.M.S.); cardoso.alc@gmail.com (A.L.C.); jmpessa@gmail.com (J.P.); 2 Institute for Interdisciplinary Research (IIIUC), University of Coimbra, Coimbra 3030-789, Portugal; 3 PhD Program in Experimental Biology and Biomedicine (PDBEB), University of Coimbra, Coimbra 3004-504, Portugal; 4 Institute of Endodontics, Faculty of Medicine, University of Coimbra, Coimbra 3000-075 Portugal; ppalma@uc.pt

**Keywords:** biocompatibility, regenerative endodontics, cytotoxicity, calcium silicate cements, PulpGuard, SCAPS

## Abstract

Background: The development of materials with bioregenerative properties is critically important for vital pulp therapies and regenerative endodontic procedures. The aim of this study was to evaluate the cytocompatibility and cytotoxicity of a new endodontic biomaterial, PulpGuard, in comparison with two other biomaterials widely used in endodontic procedures, ProRoot Mineral Trioxide Aggregate (MTA) and Biodentine. Methods: Apical papilla cells (APCs) were isolated from third molars with incomplete rhizogenesis from patients with orthodontic indication for dental extraction. Cultured APCs were incubated for 24, 48, or 72 h with different dilutions of eluates prepared from the three materials. Cellular viability, mobility, and proliferation were assessed in vitro using the Alamar Blue assay and a wound-healing test. The cells were also cultured in direct contact with the surface of each material. These were then analyzed via Scanning Electron Microscopy (SEM), and the surface chemical composition was determined by Energy-Dispersive Spectroscopy (EDS). Results: Cells incubated in the presence of eluates extracted from ProRoot MTA and PulpGuard presented rates of viability comparable to those of control cells; in contrast, undiluted Biodentine eluates induced a significant reduction of cellular viability. The wound-healing assay revealed that eluates from ProRoot MTA and PulpGuard allowed for unhindered cellular migration and proliferation. Cellular adhesion was observed on the surface of all materials tested. Consistent with their disclosed composition, EDS analysis found high relative abundance of calcium in Biodentine and ProRoot MTA and high abundance of silicon in PulpGuard. Significant amounts of zinc and calcium were also present in PulpGuard discs. Concerning solubility, Biodentine and ProRoot MTA presented mild weight loss after eluate extraction, while PulpGuard discs showed significant water uptake. Conclusions: PulpGuard displayed a good in vitro cytocompatibility profile and did not significantly affect the proliferation and migration rates of APCs. Cells cultured in the presence of PulpGuard eluates displayed a similar profile to those cultured with eluates from the widely used endodontic cement ProRoot MTA.

## 1. Introduction

The development of new materials with bioregenerative properties has been a major goal in biomaterial engineering. The use of these products on vital pulp therapies and regenerative endodontic procedures must provide a barrier for blood clot and microorganisms and simultaneously possess adequate biocompatibility and bioactive properties to induce tissue repair or stimulate stem cell differentiation and regeneration [1,2]. A major breakthrough in this field occurred 20 years ago with the introduction of Mineral Trioxide Aggregate (MTA) into clinical use. Its initial presentation was gray, however, due to handling and discoloration issues, but a white MTA (ProRoot MTA, Dentsply Tulsa, OK, USA) composition was later developed [3]. MTA is the most common biomaterial used in endodontic treatment and is primarily composed of tricalcium and dicalcium silicate with bismuth oxide (Table S1—Composition and setting time of the tested materials) [4]. Hydration of this powder results in the formation of calcium hydroxide and a calcium silicate hydrate gel which, after a period of time, transforms into a poorly crystallized and porous solid [5]. This material has consistently shown to display excellent biocompatibility properties in vitro and low cytotoxicity [6] and is associated with improved clinical outcome when used for direct pulp capping [7], pulpotomy [8], or apexification [9]. Several studies have revealed that ProRoot MTA can induce cell attachment, proliferation, and differentiation of dental pulp stem cells and apical papilla stem cells into cells that are able to produce mineralized tissue [10]. This is thought to occurs via the activation of specific genes expressed during odontoblast differentiation, such as dentin matrix acidic phosphoprotein 1 (DMP-1), dentin sialoprotein (DSPP) and matrix extracellular phosphoglycoprotein (MEPE) [11].

In 2009, a new tricalcium silicate cement, Biodentine (BD, Septodont, Saint-Maur-des-Fossés, France), became commercially available. The powder component of Biodentine consists of tricalcium and dicalcium silicate (main and second core materials), a calcium carbonate and oxide filler, iron oxide to impart shade, and zirconium oxide (as a radiopacifier). The liquid component contains calcium chloride (accelerator) and a hydrosoluble polymer (as a water-reducing agent). Biodentine also presents good biocompatibility with gingival fibroblasts [12] and dental pulp cells [13] and promotes dentine bridge/tertiary dentin formation [14]. It has also been suggested that Biodentine promotes cell differentiation and reparative dentine synthesis. Comparatively to MTA, this material provides improvements in terms of manipulation in the clinical setting as it completes its setting in 12 min, allowing restorative procedures to be performed in one-visit treatment [15], and causes less tooth discoloration at one-year evaluation [16].

Recently, a new bioactive endodontic cement has been developed under the name of PulpGuard (Coltène/Whaledent, Altstätten, Switzerland). This material includes in its chemical composition silicates, polydimethylsiloxane, silicone oils, platinum catalyst, zinc oxide, zirconium dioxide, bioactive glass, and pigment. PulpGuard has similar clinical applications to ProRoot MTA and Biodentine; however, there are currently no published studies regarding its cytotoxicity or effect on cell proliferation.

The biocompatibility of endodontic cements is extremely important as they have to be in contact with a live tissue without producing adverse effects, such as activation of a host response or direct induction of cell death [17]. As bioactive materials are assumed to interact with pulp and periapical cells directly or through diffusion of components within the living periradicular tissue, assessing their biocompatibility is critical to ascertain their potential influence on reparative and regenerative responses.

A comprehensive biocompatibility assessment implies initial screening tests to evaluate the toxicity profile in cells in direct and indirect contact [18] and secondary in vivo tests with laboratory animals [19]. Bioactive endodontic cements have been studied in different cell types, including fibroblasts and dental stem cells from various niches [10]. Apical papilla cells (APCs) are a good model for cytocompatibility tests because bioceramic materials may come in contact with these cells after clinical interventions [20]. These cells are isolated from human teeth with incomplete rhizogenesis and are known to contain fibroblasts and a significant population of stem cells of the apical papilla (SCAPs) [21]. Importantly, SCAPs have a proliferation rate 2–3 times higher than other pulp cells [21] and are considered potent stem cells in terms of their odontogenic, osteogenic, and neurogenic potential [21,22].

Cytocompatibility tests with human dental cells allow us to understand the biological value of novel materials with potential for regenerative procedures. The aim of this study was to evaluate the cytocompatibility and cytotoxicity of a new endodontic biomaterial (PulpGuard) on APCs in comparison with two widely used biomaterials (ProRoot MTA and Biodentine). The null hypothesis was that there are no difference in cytocompatibility and cytotoxicity between the selected endodontic bioceramic materials.

## 2. Materials and Methods

### 2.1. Eluate Extracts

The endodontic cements tested in this study were ProRoot MTA (DentsplyTulsa Dental, Tulsa, OK, USA), Biodentine (Septodont, Saint-Maur-des-Fossés, France), and PulpGuard (Coltène-Whaledent, Altstätten, Switzerland). The materials were prepared according to the manufacturer’s instructions, under sterile conditions. Sample discs were shaped in silicone molds, measuring 5 mm in diameter and 2 mm in thickness. After setting, the samples were sterilized under ultraviolet irradiation for 15 min. Next, to achieve a complete setting of each material, the sample discs were incubated for 48 h at 37 °C in a humidified cell culture incubator. Next, eluate extraction was performed in sterile conditions using APC culture media (Knockout-DMEM, Gibco^®^, supplemented with 20% Fetal Bovine Serum, 100 U/mL penicillin, 100 μg/mL streptomycin, Glutamax, and β-mercaptoethanol). This procedure was carried out according to International Organization for Standardization (ISO) guideline 10993-12, and the ratio of material surface area to extraction vehicle volume was calculated as 1.5 cm^2^/mL (ISO 10993-5). After 24 h, the extraction medium was centrifuged at 1000 g to remove any large particles or debris from the solution. The pH of undiluted eluates was measured at room temperature with not CO_2_ pre-incubation for the different materials using an HI9025 pH meter (HANNA Instruments, Póvoa do Varzim, Portugal). The undiluted extract media were reserved for experimental procedures, and two dilutions of 1:2 and 1:4 in APC media were prepared.

### 2.2. Isolation and Culture of APCs

Third molars with incomplete rhizogenesis were collected from patients (14–18 years of age) that presented orthodontic indication for extraction. Informed consent was obtained from all participants, according to the approval of the Ethical Committee of IRB, Faculty of Medicine, University of Coimbra (Project CE-028/2016) and following the guidelines of the Declaration of Helsinki. Upon dental extraction, the apical papilla was gently detached from the apical foramen of the root and minced into small portions. Apical tissues were then enzymatically digested with type I collagenase (3 mg/mL) and dispase (4 mg/mL) for 1 hour at 37 °C. The suspension was gently mixed every 15 min to facilitate the dissociation of the tissue. To obtain single-cell suspensions, the solution was resuspended and passed through a 70 μm cell strainer, followed by centrifugation at 300 g for 5 min, at room temperature. The cell pellet was then resuspended and cultured in APC medium (as above) at 37 °C in an incubator with 5% CO_2_. The medium was changed every three days, and the cells were cultured in T75 flasks (Corning) until achieving 80% confluence. The cells were used for downstream application between passages 2 and 5. Cells in the control condition underwent similar manipulations as those treated with the eluates.

### 2.3. Scanning Electron Microscopy (SEM) and Energy-Dispersive Spectroscopy Analysis (EDS)

Disc samples of MTA, Biodentine (BD), and PulpGuard were prepared as previously described and dispensed in 24-well plates. Cell suspensions from cultured APCs were then directly seeded over each biomaterial disc at a density of 5 × 10^4^ cells/mL. After 72 h of incubation, the samples were removed from the incubator (37 °C, 5% CO_2_) and fixed with 3% glutaraldehyde (in PBS). The sample discs were then dehydrated using increasing concentrations of ethanol (50%, 70%, 96%, and 100%) before proceeding to Scanning Electron Microscopy Analysis. The dehydrated discs were immersed in a conductive carbon cement for 24 h and coated with gold–palladium (Au–Pd). The samples were examined by SEM using a Hitachi SU-70 microscope, and the composition of each material was analyzed by Energy-Dispersive Spectroscopy Analysis (EDS).

### 2.4. Cell Viability Assay—Alamar Blue

Cell viability was determined using the Alamar Blue assay. In this test, cellular metabolic activity generates a reducing environment. In presence of viable cells, non-fluorescent Resazurin (blue) (Sigma-Aldrich #R7017, Sintra, Portugal) is reduced to a red, fluorescent resorufin, increasing the overall fluorescence and red color of the culture medium.

For this test, APCs were seeded in 96-well culture plates at a density of 1 × 10^4^ cells/well. Material extracts were then added, and the cells were incubated at 37 °C in a 5% CO_2_ atmosphere for 24, 48, or 72 h. At the end of each incubation time, cell viability was assessed by adding the Alamar Blue reagent 10% (v/v) to the culture medium at a ratio of 1:200. The cells were kept in culture for 4–5 h at 37 °C to allow reduction of the agent. Following this period, absorbance values were read in a plate spectrophotometer (Spectra Max Plus 384, Molecular Devices, Silicon Valley, CA, USA) at 570 nm and 600 nm. Cell viability was calculated as a percentage of control cells’ absorbance, using the formula: (A_570_ − A_600_) of treated cells × 100/(A_570_ − A_600_) of control cells. Three independent replicates were used per condition, and each experiment was performed in duplicate. The results are presented as percentage of cell viability with respect to control for each condition tested.

### 2.5. Wound-Healing Assay

Cell migration and proliferation were evaluated using a test specifically developed for “wound-healing” analysis (ibidi GmbH, Martinsried, Germany). APCs were seeded in the wells of inserts at a concentration of 5 × 10^5^ cells/mL and allowed to grow in the designated areas for 24 h. After this pre-incubation, the culture insert was removed under sterile conditions to generate a gap of 500 µm in the culture. The healing process was allowed to proceed in the absence (control group) or in the presence of undiluted biomaterial eluates. Observation of the “wound” was performed at 0 h, 24 h, and 48 h. The results are expressed as percentage of healed “wound”, quantified using ImageJ Software, assessed as the percentage of open area in the original field of view.

### 2.6. Solubility Test

The solubility of the three endodontic cements was assessed using 21–26 discs/material. For this purpose, the discs were cured for 48 h in an incubator at 37 °C, 5% CO_2_, in a humidified ambient. After achieving complete setting, each sample was weighed three times (Initial Weight (I_W_)) and then stored in culture medium for 24 h at 37 °C. After this period of immersion, the discs were removed from the medium, dried to remove the excess of liquid, and stored at 37 °C until the weight was stable. At this point, using an analytic balance, each sample was weighted three times (Final Weight (F_W_)). Solubility (S) was calculated as the percentage of the initial weight (S = ((I_W_ − F_W_)/F_W_) × 100).

### 2.7. Statistical Analysis

For the cell viability assay, one-way ANOVA (comparing different dilutions) or two-way ANOVA (comparing different compounds over incubation time) were used to analyze the effects of the material, eluate dilution, and exposure time, as indicated in individual figure legends. Post-hoc comparisons were performed using a Dunnett´s post-hoc test for multiple comparisons. One-way ANOVA with Dunnett’s post-hoc test was used to examine the wound-healing assay. In the solubility test, the results were analyzed by one-sample t-test with the null hypothesis that the materials should not gain or lose weight after incubation (theoretical mean of 0% weight change). All data were analyzed using GraphPad Prism Version software. All data are presented as means ± standard error of the mean and correspond to at least three independent biological replicates unless stated otherwise; statistical significance was set to * *p* < 0.05, ** *p* < 0.01, and *** *p* < 0.001.

## 3. Results

**Figure 1:**
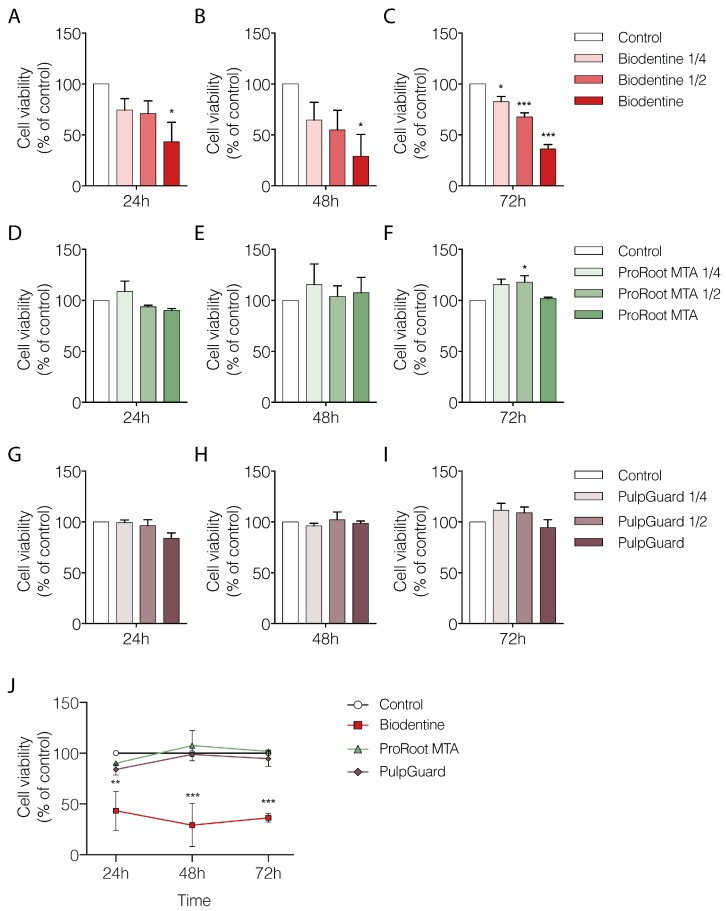
Viability of apical papilla cells incubated in the presence of different endodontic cement eluates. (**A**–**I**) Cells were grown in the presence of (**A**–**C**) Biodentine, (**D**–**F**) ProRoot Mineral Trioxide Aggregate (MTA), or (**G**–**I**) PulpGuard eluates at different concentrations (undiluted, 1:2 dilution, and 1:4 dilution). Cellular viability was assess using the Alamar Blue method at 24 h (**A**,**D**,**G**), 48 h (**B**,**E**,**H**), or 72 h (**C**,**F**,**I**). (**J**) Relative cytotoxicity values of undiluted biocements were compared across time and showed that Biodentine significantly altered the cell viability profile when compared to ProRoot MTA and PulpGuard. These two endodontic cements showed similar influence on cell viability when compared to control conditions. The values for each experiment were normalized to the average of the control cells (not exposed to cement eluate). Results are from n = 3, presented as means ± SEM. Significative differences are indicated as * *p* < 0.05, ** *p* < 0.01, and *** *p* < 0.001; One-way ANOVA with Dunnett’s multiple comparisons test was used in (**A**–**I**); two-way ANOVA with Dunnett’s multiple comparisons test was used in (**J**).

We used the Alamar Blue assay to analyze cellular metabolism and viability. We performed measurements of APCs cultured for 24, 48, or 72 h in the presence of varying dilutions of each biomaterial eluate (undiluted, 1:2, and 1:4 dilutions). The results presented in Figure 1 indicate the percentage of cellular viability when APCs were grown in the presence of different concentrations of the biomaterial eluates, normalized to the control group (treated with culture media only). Our results show that undiluted Biodentine eluates significantly affected APC viability already at the 24 h time point (42.2 ± 19.19%; *p* < 0.05; Figure 1A). The same trend was also seen after 48 or 72 h of incubation, where there was a linear dose–response relationship for the eluate concentrations tested (Figure 1B,C). At the 72 h time point, undiluted Biodentine eluates reduced cell viability to 36.4 ± 4.22% (*p* < 0.001) when compared to control (Figure 1C). Moreover, even the most diluted Biodentine eluate (1:4 dilution) produced a statistically significant reduction in cellular viability after 72 h (82.7 ± 4.99%; *p* < 0.05).

Our results for ProRoot MTA showed no significant deleterious effect on cellular viability, regardless of the time point or concentration used (Figure 1D–F). Interestingly, at the 72 h, there was a small but significant increase in viability for cells grown in the presence of MTA eluate at 1:2 dilution (117.9 ± 6.25%; *p* < 0.05; Figure 1F). This effect suggests there was a non-linear dose-dependent response only when reduced amounts of eluate were present. Nevertheless, the undiluted MTA eluate at the 72 h maintained a cellular viability level similar to that in control conditions (101.7 ± 1.36; *p* = 0.98).

Regarding the effects of PulpGuard, there was no significant alteration in cellular viability regardless of incubation time or concentrations used, suggesting this material is well tolerated by APCs (Figure 1G–I). Indeed, for the longest exposure (72 h) and highest concentration (undiluted PulpGuard), cellular viability was 94.5 ± 7.60% compared to control conditions (*p* = 0.85).

Taken together, our results indicate that when comparing only the most concentrated eluates across time and between different biomaterials, only Biodentine shows a significant reduction in cellular viability compared to the control conditions or to other endodontic cements (Two-way ANOVA: Effect of compound *p* < 0.001) (Figure 1J and Table 1).

**Figure 2:**
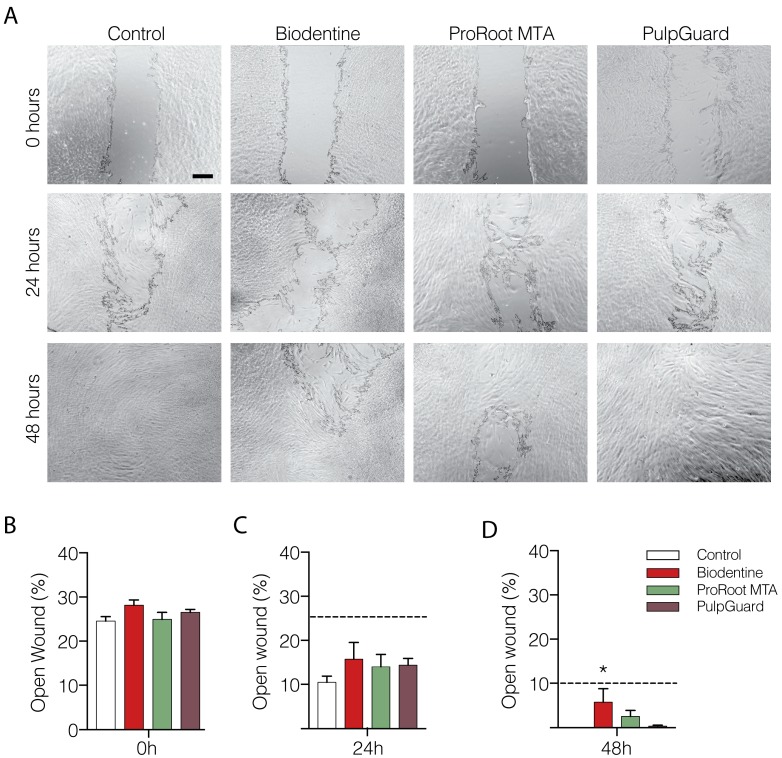
Effect of endodontic cement eluates on cellular migration and proliferation in a wound-healing assay. (**A**) Representative images of wound closure at the initial time point (0 h), 24, or 48 h after the removal of a cell culture insert and when apical papilla cells were grown in the presence of undiluted eluate extracts; scale bar 250 µm. (**B**) Following the removal of the wound-generating insert, the percentage of open wound was assessed for Biodentine (red), ProRoot MTA (green), PulpGuard (purple), and control conditions (white). (**C**,**D**) Wound closure was re-assessed following 24 h (**C**) and 48 h (**D**); the dotted line indicates the percentage of open wound for the control conditions. The results are from n = 4–7, presented as means ± SEM. Significative differences are indicated as * *p* < 0.05; One-way ANOVA with Dunnett’s multiple comparisons test was used in (**B**,**C**)

We also performed a “wound-healing” assay to assess proliferation and mobility of APCs exposed to Biodentine, ProRoot MTA, and PulpGuard eluates. The wound area was determined after the removal of the scarring insert, and the percentage of open wound was determined in the following 24 and 48 h (Figure 2A). Our results show that after 24 h, wound healing progressed in all experimental conditions with only a slight deceleration in cells exposed to the Biodentine eluate (*p* < 0.05). After 48 h, the wound was closed in control conditions, whereas a small percentage of the plate remained exposed in the other experimental conditions, particularly for Biodentine (Figure 2A,C). Among the three materials, PulpGuard seemed to interfere the least with the ability of APCs to cover the wound revealed after the removal of the cell culture insert.

**Figure 3:**
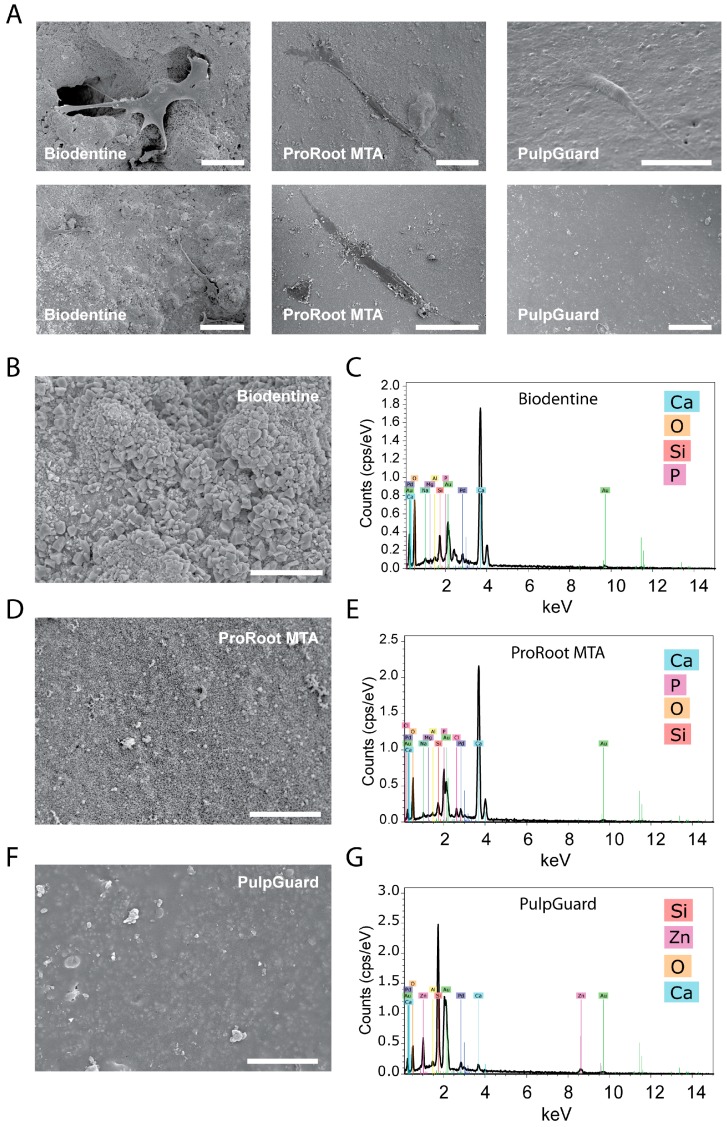
Surface properties and composition of biomaterials under scanning electron microscopy and energy-dispersive spectroscopy (EDS) analysis. (**A**) Representative images at different magnifications of the surface characteristics of Biodentine (left), ProRoot MTA (center), and PulpGuard (right), scale bar = 100 µm. (**B**,**C**) High-magnification image of the surface characteristics of Biodentine cement (**B**) revealing a rough surface; EDS analysis (**C**) displaying the presence of relative high amounts of calcium, oxygen, silicon, and phosphorous; scale bar = 10 µm. (**D**,**E**) High-magnification image of the surface characteristics of ProRoot MTA (**D**) revealing a rough and porous surface; EDS analysis (**E**) displaying the presence of relative high amounts of calcium, phosphorous, oxygen, and silicon; scale bar = 10 µm. (**F**,**G**) High-magnification image of the surface characteristics of PulpGuard (**F**) revealing a mostly smooth surface with little porosity; EDS analysis (**G**) displaying the presence of relative high amounts of silicon, zinc, oxygen, and calcium; scale bar = 10 µm.

Direct observation of the biomaterials via SEM allowed us to identify and assess cell morphology when they were present on the material surface. As shown in Figure 3, we found cells on top of all materials. These cells were well individualized, flattened, and spindle-like in shape, with multiple prolongations.

The materials tested showed different surface morphologies. ProRoot MTA and Biodentine showed a porous and rough surface, while PulpGuard displayed a smooth, uniform, and apparently homogeneous microstructure with some major surface cracks. Biodentine exhibited polygonal particles on the surface, while smaller irregular particles were visible on ProRoot MTA surface. PulpGuard showed crystalline structures in close proximity to the surface cracks. We also used EDS as an analytic technique for the chemical characterization and identification of the major elements present in each disc samples. As displayed in Figure 3B–D, EDS indicated there is high abundance of calcium in Biodentine and ProRoot MTA, while the prevalent element in PulpGuard is silicon. This material also presents significant amounts of calcium and zinc.

**Figure 4:**
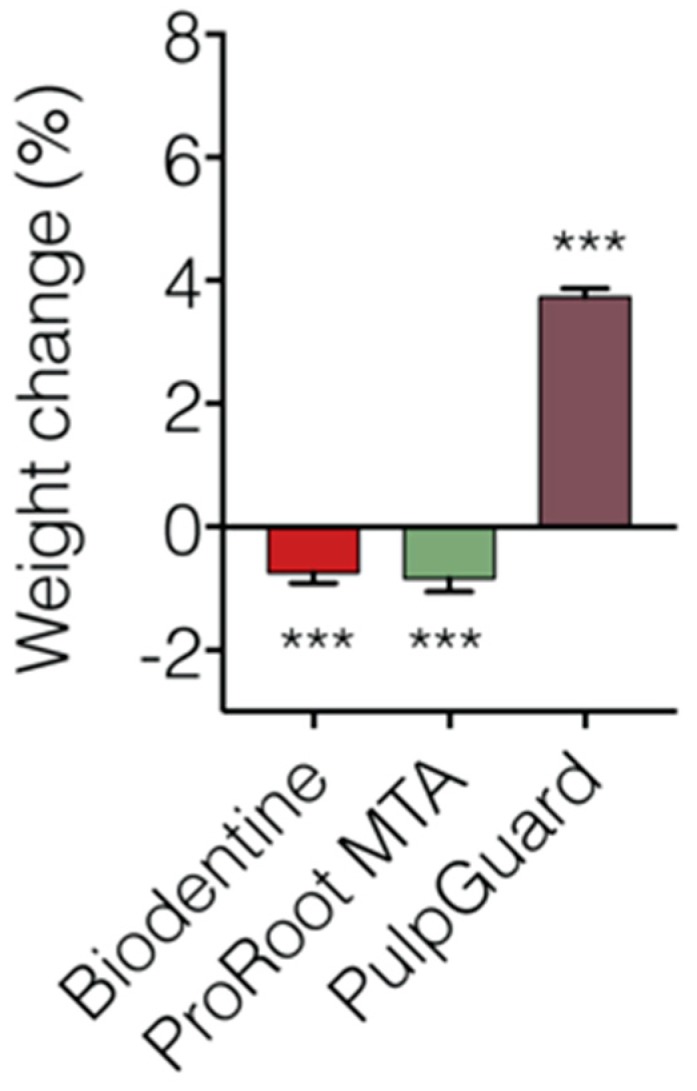
Relative solubility properties of the individual biomaterials. Weight change in sample discs following incubation of the different materials for 48 h in cell culture medium. The results are from n = 21–26, presented as means ± SEM. Significative differences are indicated as *** *p* < 0.001; One-sample t-test was used to determine statistically significant changes in weight.

We also performed a solubility assay using cell culture medium to simulate biological fluids. The solubility index of the three materials is presented in Figure 4 and Table 2. Concerning this test, our reports show that Biodentine and ProRoot MTA lost significant weight after immersion for 48 h. The Biodentine discs presented a weight loss of –0.73 ± 0.174%. ProRoot MTA was slightly more soluble, presenting a final weight of –0.84 ± 0.220%. PulpGuard showed to be slightly hygroscopic, presenting a significant final weight gain of 3.74 ± 0.130%. In terms of pH following eluate extraction, we determined that Biodentine pH was 7.57 ± 0.01, ProRoot MTA pH was 8.02 ± 0.03, and PulpGuard pH was 8.43 ± 0.03. While still within physiological values, ProRoot MTA and PulpGuard displayed slightly more alkaline pH than Biodentine.

## 4. Discussion

Evaluating the cytotoxicity of new endodontic cements is critical because of the impact these materials may have on viability, proliferation, and differentiation of diverse types of dental stem cells. In the present work, we used well-established cell culture assays to compare the effects on APCs of three endodontic cements. We tested two widely used calcium silicate-based cements, Biodentine and ProRoot MTA, and a new bioceramic, silicone-based cement, PulpGuard.

We selected to study the effects of these three materials in assays involving direct and indirect contact of the materials with cells of the apical papilla. It is important to mention that the apical papilla contains a significant population of stem cells that hold superior regenerative potential [23]. The particular response of stem cells to different materials may be a concern for applications relying on bioactive cements [24].

Biomaterials in contact with media or biological fluids not only release particles into solution but may also suffer from alterations that lead to changes in the features of the materials itself [25,26]. In fact, cell proliferation and attachment can be affected by water and protein adsorption when the material is in contact with the media [25,26]. Our results show that both ProRoot MTA and Biodentine presented significant solubility, suggesting that any water uptake would not compensate for material loss during the extraction of the eluates. Conversely, discs prepared with PulpGuard presented significant weight gain, suggesting there is a significant water uptake by this material. This is consistent with PulpGuard presenting a composition and biophysical characteristics that are similar to other cements that contain bioactive glass, such as GuttaFlow bioseal, which is known to display high water absorption and low solubility [27].

Our results from the cellular viability assays found that ProRoot MTA and PulpGuard presented high cytocompatibility and low toxicity as evidenced by the Alamar Blue assay which measures the reducing potential of cellular metabolism. It is also noteworthy that longer incubations with diluted ProRoot MTA gave rise to a small, but statistically significant increase in cellular viability. These results are consistent with published data showing that MTA may induce an increase in proliferation of specific types of dental cells [28]. Interestingly, PulpGuard showed a similar trend towards increased viability.

Interestingly, we found that Biodentine eluates showed low-to-moderate effects on cellular viability and in the wound-healing assay. This is consistent with previous reports describing that high concentrations of Biodentine in the cell culture medium significantly decrease stem cell proliferation [6,13]. One possible explanation for this observation is that Biodentine is described to enhance the differentiation of stem cells [29]. This is interesting not only because this property may confound results that are dependent on cellular proliferation, but also because it may present benefits regarding a regenerative potential of the material. Therefore, future studies should aim to distinguish between bioactive properties that influence differentiation, since these may indirectly impact on proliferation and, as such, interfere with most measures of cellular viability. It would also be interesting to assess if bioactive compounds affect mitochondrial activity and to perform measures of cellular viability in quiescent or non-proliferating cells. In our case, since APCs contain a substantial population of SCAPs which display strong growth potential, these may be strongly influenced by agents that induce cellular differentiation.

Cell adhesion is another good indicator of cytocompatibility, and it has been indicated that biocompatible materials often preserve cellular adherence [30]. Scanning electron microscopy is therefore valuable to assess the behavior of cells in direct contact with biomaterials. Nevertheless, there are drawbacks that have been reported in relation to the observation of calcium silicate-based materials [31]. In particular, sample preparation for SEM includes fixation and dehydration which may lead to chemical and surface alterations, and these in turn may confound a reliable observation of the samples. In our study, we found APCs adhered to discs prepared from all three materials. Differences in the degree of surface roughness and morphology, chemical composition, material bioactivity, and material microstructure are known to influence cellular adhesion. For example, increased roughness and porosity has been correlated with increase cell adhesion [32,33]. Recent studies have reported that spindle-shaped cells observed in contact with biomaterials such as Biodentine are a good indicator of low-toxicity of the materials [30]. Biodentine and MTA have both been reported to promote the adhesion of pulp stem cells and human gingival fibroblasts [12,13]. However, further studies using more quantitative methods are required to assess if the surface properties of PulpGuard may contribute or inhibit cellular adhesion in comparison to other cements.

We analyzed the chemical composition of the surface of the different materials by energy dispersive spectroscopy. Our results showed a similar composition for ProRoot MTA and Biodentine. These materials are composed of tricalcium silicate, dicalcium silicate, tricalcium aluminate, tetracalcium aluminoferrite with bismuth oxide as radiopacifier for ProRoot MTA [34], and tricalcium silicate, calcium carbonate, and zirconium oxide as a radiopacity agent for Biodentine [35]. According to the manufacture’s information, PulpGuard is composed of silicates, polydimethylsiloxane, silicone oils, zinc oxide, platinum catalyst, zirconium dioxide, and bioactive glass (Coltène/Whaledent, Altstätten, Switzerland). Both ProRoot MTA and Biodentine revealed the presence of calcium, silicon, oxygen, and phosphate, which is in accordance with a recent study looking at the composition of these materials [36]. PulpGuard, on the other hand, revealed relative high abundance of silicon, zinc, and oxygen, and relative lower abundance of calcium, which is compatible with the manufacturer’s indications. Importantly, previous studies have found that the presence of Zn^2+^ in culture is correlated with cytotoxic properties [37]; however, the low toxicity presented by PulpGuard under our experimental conditions, suggests that the potential leaching of this ionic species is not sufficient to promote significant cytotoxicity. On the contrary, we observed a trend, although not statistically significant, for enhanced proliferation when diluted eluates were present in culture for longer durations. For ProRoot MTA, these effects are well documented and may be due to the presence of calcium silicate-based materials [38].

We also measured the solubility of the endodontic sealers. Our results combined multiple factors, such as the potential loss of material via disintegration, solubilization during extraction, as well as weight gain via water uptake and material adsorption (e.g., protein) to the sample. Importantly, sample disintegration and solubilization may compensate water uptake during the immersion test, but these effects may also occur simultaneously with dimensional changes of the biomaterials [39,40,41]. To enhance accuracy and simulate the effects under biological fluids, our materials were immersed only once in cell culture media. No discs were re-used to avoid stress effects from repeated drying and immersion and to avoid depletion of soluble material from repeated extractions. We found that after 48 h, both Biodentine and ProRoot MTA exhibited a slight weight loss in culture media, which is in agreement with results testing the water solubility of these compounds [42]; however, here we did not test the long-term solubility of either material. Calcium silicate cements exhibit the formation of calcium hydroxide during the early stage of the setting process. The release of OH^−^ and Ca^2+^ ions is related to the solubility of the biomaterials and their antimicrobial properties. Other studies have also mentioned that calcium ions can react with phosphate-containing liquids and form hydroxyapatite on the surface [43]; however, no hydroxyapatite crystals were observed on the surface of ProRoot MTA and Biodentine in our study. To the best of our knowledge, no data concerning the solubility of PulpGuard cement are presently available. In our study, this biomaterial presented a significant final weight gain, which may be due to a combination of greater resistance to material leaching and degradation, as well as to significant water uptake. These factors may influence the bioactivity of the material.

At scanning electron microscopic observation, we identified the presence of crystals near structural fissures. EDM analysis determined that these crystals were rich in sodium.

## 5. Conclusions

The present study allows us to conclude, within some limitations, that ProRoot MTA and PulpGuard display good cytocompatibility with human cells from the apical papilla. PulpGuard did not significantly affect cell proliferation or migration rates. Under electron microscopy, cellular adhesion was visible in direct contact with all three materials. Further experiments supported by in vivo models are necessary to validate the data acquired in this study and to investigate if these materials, particularly PulpGuard, have any effects on cell differentiation and tissue regeneration.

## Figures and Tables

**Table 1:** Statistical analysis of cellular viability over time.

Source of Variation	% of Total Variation	*p* Value	*p* Value	Significant?	*p* Value
Interaction	2.886	0.7601	ns	No	
Time point	0.426	0.7835	ns	No	
Biomaterial	75.95	<0.0001	***	Yes	
ANOVA table	SS	DF	MS	F(DFn,DFd)	*p* value
Interaction	970.8	6	161.8	F(6,24) = 0.5567	*p* = 0.7601
Row Factor	143.3	2	71.65	F(2,24) = 0.2465	*p* = 0.7835
Biomaterial	25,549	3	8516	F(3,24) = 29.3	*p* < 0.0001
Residual	6976	24	290.7		

SS–Sum of Squares; DF–Degrees of freedoms; MS–Mean squares.

**Table 2:** Statistical analysis of the solubility test.

Analysis	Biodentine	ProRoot MTA	PulpGuard
Number of values (n)	21	26	26
Mean (%)	−0.735	−0.8366	3.74
Std. Error of Mean	0.1743	0.22	0.1296
One sample t test			
Theoretical mean	0	0	0
Actual mean	−0.735	−0.8366	3.74
Discrepancy	−0.735	−0.8366	3.74
95% CI of discrepancy	−1.099 to −0.3715	−1.29 to −0.3836	3.473 to 4.007
t, df	t = 4.217 df = 20	t = 3.803 df = 25	t = 28.85 df = 25
*p* value (two tailed)	0.0004	0.0008	<0.0001

## Supplementary Materials

The following are available online at http://www.mdpi.com/2079-4983/9/4/74/s1, Table S1: Composition and setting time of tested materials.

## References

[1] Gandolfi M.G., Spagnuolo G., Siboni F., Procino A., Rivieccio V., Pelliccioni G.A., Prati C., Rengo S. (2015). Calcium silicate/calcium phosphate biphasic cements for vital pulp therapy: Chemical-physical properties and human pulp cells response. Clin. Oral Investig..

[2] Rodríguez-Lozano F.J., García-Bernal D., Oñate-Sánchez R.E., Ortolani-Seltenerich P.S., Forner L., Moraleda J.M. (2017). Evaluation of cytocompatibility of calcium silicate-based endodontic sealers and their effects on the biological responses of mesenchymal dental stem cells. Int. Endod. J..

[3] Parirokh M., Torabinejad M. (2010). Mineral trioxide aggregate: a comprehensive literature review—Part I: Chemical, physical, and antibacterial properties. J. Endod..

[4] Camilleri J., Montesin F.E., Brady K., Sweeney R., Curtis R.V., Ford T.R.P. (2005). The constitution of mineral trioxide aggregate. Dent. Mater..

[5] Camilleri J. (2007). Hydration mechanisms of mineral trioxide aggregate. Int. Endod. J..

[6] Küçükkaya S., Görduysus M.Ö., Zeybek N.D., Müftüoğlu S.F. (2016). In Vitro Cytotoxicity of Calcium Silicate-Based Endodontic Cement as Root-End Filling Materials. Scientifica.

[7] Brizuela C., Ormeño A., Cabrera C., Cabezas R., Silva C.I., Ramírez V., Mercade M. (2017). Direct Pulp Capping with Calcium Hydroxide, Mineral Trioxide Aggregate, and Biodentine in Permanent Young Teeth with Caries: A Randomized Clinical Trial. J. Endod..

[8] Taha N.A., Ahmad M.B., Ghanim A. (2017). Assessment of Mineral Trioxide Aggregate pulpotomy in mature permanent teeth with carious exposures. Int. Endod. J..

[9] Mente J., Leo M., Panagidis D., Ohle M., Schneider S., Bermejo J.L., Pfefferle T. (2013). Treatment outcome of mineral trioxide aggregate in open apex teeth. J. Endod..

[10] Emara R., Elhennawy K., Schwendicke F. (2018). Effects of calcium silicate cements on dental pulp cells: A systematic review. J. Dent..

[11] Lee S.K., Lee S.K., Lee S.I., Park J.H., Jang J.H., Kim H.W., Kim E.C. (2010). Effect of calcium phosphate cements on growth and odontoblastic differentiation in human dental pulp cells. J. Endod..

[12] Zhou H.M., Shen Y., Wang Z.J., Li L., Zheng Y.F., Häkkinen L., Haapasalo M. (2013). In vitro cytotoxicity evaluation of a novel root repair material. J. Endod..

[13] Luo Z., Li D., Kohli M.R., Yu Q., Kim S., He W.X. (2014). Effect of Biodentine on the proliferation, migration and adhesion of human dental pulp stem cells. J. Dent..

[14] Nowicka A., Lipski M., Parafiniuk M., Sporniak-Tutak K., Lichota D., Kosierkiewicz A., Kaczmarek W., Buczkowska-Radlińska J. (2013). Response of human dental pulp capped with biodentine and mineral trioxide aggregate. J. Endod..

[15] Palma P., Marques J., Falacho R., Vinagre A., Santos J., Ramos J. (2018). Does Delayed Restoration Improve Shear Bond Strength of Different Restorative Protocols to Calcium Silicate-Based Cements?. Materials.

[16] Ramos J.C., Palma P.J., Nascimento R., Caramelo F., Messias A., Vinagre A., Santos J.M. (2016). 1-Year In Vitro Evaluation of Tooth Discoloration Induced by 2 Calcium Silicate-based Cements. J. Endod..

[17] Williams D. (2003). Revisiting the definition of biocompatibility. Med. Device Technol..

[18] Tomás-Catalá C.J., Collado-González M., García-Bernal D., Oñate-Sánchez R.E., Forner L., Llena C., Lozano A., Moraleda J.M., Rodríguez-Lozano F.J. (2018). Biocompatibility of New Pulp-capping Materials NeoMTA Plus, MTA Repair HP, and Biodentine on Human Dental Pulp Stem Cells. J. Endod..

[19] Santos J.M., Pereira S., Sequeira D., Messias A., Martins J., Cunha H., Palma P.J., Santos A.C. (2019). Biocompatibility of a bioceramic silicon-based sealer in subcutaneous tissue. J. Oral Sci..

[20] Wongwatanasanti N., Jantarat J., Sritanaudomchai H., Hargreaves K.M. (2018). Effect of Bioceramic Materials on Proliferation and Odontoblast Differentiation of Human Stem Cells from the Apical Papilla. J. Endod..

[21] Sonoyama W., Liu Y., Yamaza T., Tuan R.S., Wang S., Shi S., Huang G.T.J. (2008). Characterization of the apical papilla and its residing stem cells from human immature permanent teeth: A pilot study. J. Endod..

[22] Bakopoulou A., Leyhausen G., Volk J., Tsiftsoglou A., Garefis P., Koidis P., Geurtsen W. (2011). Comparative analysis of in vitro osteo/odontogenic differentiation potential of human dental pulp stem cells (DPSCs) and stem cells from the apical papilla (SCAP). Arch. Oral Biol..

[23] Sonoyama W., Liu Y., Fang D., Yamaza T., Seo B.M., Zhang C., Liu H., Wang S. (2006). Mesenchymal stem cell-mediated functional tooth regeneration in swine. PLoS One.

[24] Shuai Y., Ma Y., Guo T., Zhang L., Yang R., Qi M., Liu W., Jin Y. (2018). Dental Stem Cells and Tooth Regeneration. Adv. Exp. Med. Biol..

[25] Wilson C.J., Clegg R.E., Leavesley D.I., Pearcy M.J. (2005). Mediation of biomaterial-cell interactions by adsorbed proteins: A review. Tissue Eng..

[26] Schmalz G., Galler K.M. (2017). Biocompatibility of biomaterials—Lessons learned and considerations for the design of novel materials. Dent. Mater..

[27] Gandolfi M.G., Siboni F., Prati C. (2016). Properties of a novel polysiloxane-guttapercha calcium silicate-bioglass-containing root canal sealer. Dent. Mater..

[28] Moghaddame-Jafari S., Mantellini M.G., Botero T.M., McDonald N.J., Nör J.E. (2005). Effect of ProRoot MTA on pulp cell apoptosis and proliferation in vitro. J. Endod..

[29] Zanini M., Sautier J.M., Berdal A., Simon S. (2012). Biodentine induces immortalized murine pulp cell differentiation into odontoblast-like cells and stimulates biomineralization. J. Endod..

[30] Akbulut M.B., Uyar Arpaci P., Unverdi Eldeniz A. (2016). Effects of novel root repair materials on attachment and morphological behaviour of periodontal ligament fibroblasts: Scanning electron microscopy observation. Microsc. Res. Tech..

[31] Camilleri J., Montesin F.E., Papaioannou S., McDonald F., Pitt Ford T.R. (2004). Biocompatibility of two commercial forms of mineral trioxide aggregate. Int. Endod. J..

[32] Butt N., Talwar S., Chaudhry S., Nawal R., Yadav S., Bali A. (2014). Comparison of physical and mechanical properties of mineral trioxide aggregate and Biodentine. Indian J. Dent. Res..

[33] Shi W., Mozumder M.S., Zhang H., Zhu J., Perinpanayagam H. (2012). MTA-enriched nanocomposite TiO_2_-polymeric powder coatings support human mesenchymal cell attachment and growth. Biomed. Mater..

[34] Camilleri J. (2008). The chemical composition of mineral trioxide aggregate. J. Conserv. Dent..

[35] Malkondu O., Karapinar Kazandag M., Kazazoglu E. (2014). A review on biodentine, a contemporary dentine replacement and repair material. Biomed. Res. Int..

[36] Gong V., Franca R. (2017). Nanoscale chemical surface characterization of four different types of dental pulp-capping materials. J. Dent..

[37] Lee J.H., Lee H.H., Kim K.N., Kim K.M. (2016). Cytotoxicity and anti-inflammatory effects of zinc ions and eugenol during setting of ZOE in immortalized human oral keratinocytes grown as three-dimensional spheroids. Dent. Mater..

[38] Kao C.T., Huang T.H., Chen Y.J., Hung C.J., Lin C.C., Shie M.Y. (2014). Using calcium silicate to regulate the physicochemical and biological properties when using beta-tricalcium phosphate as bone cement. Mater. Sci. Eng. C Mater. Biol. Appl..

[39] Orstavik D. (1983). Weight loss of endodontic sealers, cements and pastes in water. Scand. J. Dent. Res..

[40] Silva E.J., Perez R., Valentim R.M., Belladonna F.G., De-Deus G.A., Lima I.C., Neves A.A. (2017). Dissolution, dislocation and dimensional changes of endodontic sealers after a solubility challenge: A micro-CT approach. Int. Endod. J..

[41] Kazemi R.B., Safavi K.E., Spangberg L.S. (1993). Dimensional changes of endodontic sealers. Oral Surg. Oral Med. Oral Pathol..

[42] Singh S., Podar R., Dadu S., Kulkarni G., Purba R. (2015). Solubility of a new calcium silicate-based root-end filling material. J. Conserv. Dent..

[43] Kaup M., Schafer E., Dammaschke T. (2015). An in vitro study of different material properties of Biodentine compared to ProRoot MTA. Head Face Med..

