# Supplementary material for: Effects of a New Bioceramic Material on Human Apical Papilla Cells
Source: J Funct Biomater. 2018 Dec 16;9(4):74. doi: 10.3390/jfb9040074 (PMC6306901; doi:10.3390/jfb9040074)
Supplement: Supplementary file 1 [file jfb-09-00074-s001.pdf]

materials

Supplementary information

Effects of a New Bioceramic Material on Human Apical Papilla Cells

Diana B. Sequeira, Catarina M. Seabra, Paulo J. Palma, Ana Luísa Cardoso, João Peça and João Miguel Santos

Table S1. Composition and setting time of tested materials.

Materials	Manufacturer	Composition	Setting Time (minutes)
ProRoot MTA	Dentsply Tulsa, Oklahoma, USA	Powder: Tricalcium Silicate (Ca_3SiO_5), Dicalcium Silicate (Ca_2SiO_4), Bismuth Oxide and Calcium Sulfate Dehydrate Liquid: Distilled Water	165
Biodentine	Septodont, Saint-Maur-des-Fossés, France	Powder: Tricalcium Silicate (Ca_3SiO_5), Dicalcium Silicate (Ca_2SiO_4), Calcium Carbonate ($CaCO_3$), Iron Oxide (Fe_2O_3) and Zirconium Oxide (ZrO_2). Liquid: Hydrosoluble polymer and Calcium Chloride	12
PulpGuard	Coltène/Whaledent, Altstätten, Switzerland	Silicates, Polydimethylsiloxane, Silicone Oils, Platinum Catalyst, Zinc Oxide, Zirconium Dioxide, Bioactive Glass and Pigment.	3
